# Outcome prediction in pediatric fever in neutropenia: Development of clinical decision rules and external validation of published rules based on data from the prospective multicenter SPOG 2015 FN definition study

**DOI:** 10.1371/journal.pone.0287233

**Published:** 2023-08-02

**Authors:** Marina Santschi, Roland A. Ammann, Philipp K. A. Agyeman, Marc Ansari, Nicole Bodmer, Eva Brack, Christa Koenig

**Affiliations:** 1 Department of Pediatrics, Inselspital, Bern University Hospital, University of Bern, Bern, Switzerland; 2 Pediatric Hematology/Oncology, Department of Pediatrics, Inselspital, Bern University Hospital, University of Bern, Bern, Switzerland; 3 Kinderaerzte KurWerk, Burgdorf, Switzerland; 4 Pediatric Infectiology, Department of Pediatrics, Inselspital, Bern University Hospital, University of Bern, Bern, Switzerland; 5 Pediatric Hematology/Oncology, Department of Women, Child and Adolescent, University Hospital of Geneva, Geneva, Switzerland; 6 Department of Pediatrics, Gynecology, and Obstetrics, Cansearch Research Platform of Pediatric Oncology and Hematology, Faculty of Medicine, University of Geneva, Geneva, Switzerland; 7 Pediatric Oncology, University Children’s Hospital of Zürich, University of Zürich, Zürich, Switzerland; National Cancer Institute, UNITED STATES

## Abstract

**Background:**

Fever in neutropenia (FN) remains a serious complication of childhood cancer therapy. Clinical decision rules (CDRs) are recommended to help distinguish between children at high and low risk of severe infection. The aim of this analysis was to develop new CDRs for three different outcomes and to externally validate published CDRs.

**Procedure:**

Children undergoing chemotherapy for cancer were observed in a prospective multicenter study. CDRs predicting low from high risk infection regarding three outcomes (bacteremia, serious medical complications (SMC), safety relevant events (SRE)) were developed from multivariable regression models. Their predictive performance was assessed by internal cross-validation. Published CDRs suitable for validation were identified by literature search. Parameters of predictive performance were compared to assess reproducibility.

**Results:**

In 158 patients recruited between April 2016 and August 2018, 360 FN episodes were recorded, including 56 (16%) with bacteremia, 30 (8%) with SMC and 72 (20%) with SRE. The CDRs for bacteremia and SRE used four characteristics (type of malignancy, severely reduced general condition, leucocyte count <0.3 G/L, bone marrow involvement), the CDR for SMC two characteristics (severely reduced general condition and platelet count <50 G/L). Eleven published CDRs were analyzed. Six CDRs showed reproducibility, but only one in both sensitivity and specificity.

**Conclusions:**

This analysis developed CDRs predicting bacteremia, SMC or SRE at presentation with FN. In addition, it identified six published CDRs that show some reproducibility. Validation of CDRs is fundamental to find the best balance between sensitivity and specificity, and will help to further improve management of FN.

## Introduction

Fever in neutropenia (FN) remains a serious complication of childhood cancer therapy. Despite improved medical treatment, FN is still associated with significant morbidity and mortality [1]. Children receiving chemotherapeutic treatment are therefore admitted to the hospital, once they present with FN. Subsequently, they experience potentially long hospital admissions for antimicrobial treatment. Existing data supports safe and effective use of risk-stratified early discharge [2, 3] and current international guidelines for pediatric FN recommend that centers “adopt a validated risk stratification strategy and incorporate it into routine clinical management” [4].

Only a few CDRs have been published for the prediction of FN with complications in patients undergoing chemotherapy for cancer. Among these is a score predicting FN with adverse outcome from data of the same prospective multicenter study [5], data used here to predict outcomes in patients presenting with FN. More than 30 different clinical decision rules (CDRs) have been published for the prediction of complications in patients presenting with FN [6–8]. Specifically they predict bacteremia, different combinations of microbiologically or clinically defined infections or other clinical adverse outcomes, such as severe sepsis, admission to intensive care unit (ICU), or death. Before a CDR can be implemented, it must undergo validation to determine adaptability to the actual circumstances and population. While some reproducibility can be shown in most of the externally validated CDRs [9], the majority of external validations result in a lower sensitivity compared to the derivation studies. Realistic expectation of a CDRs’ predictive performance is important to identify the CDRs’ limitations and protect against missed serious infections and adverse events. The benefits of a correctly applied, validated CDR to identify “low risk” FN episodes would be a shorter time of hospitalization, and thus an increase in quality of life, reduced health costs and a decrease of the risk for nosocomial and secondary infections.

In this analysis, we used the dataset of a prospective multicenter study [10] to 1.) develop CDRs for bacteremia, serious medical complications (SMC) and safety relevant events (SRE), 2.) assess their predictive performance by internal cross-validation, and 3.) externally validate published pediatric FN CDRs and to compare their predictive performance to the respective derivation studies.

## Methods

### Design of the underlying study

The prospective multicenter Swiss Paediatric Oncology Group (SPOG) 2015 FN definition study was run in six out of nine pediatric oncology centers in Switzerland from April 2016 to August 2018 [10]. Data was collected and managed using REDCap electronic data capture tools [11]. Legal guardians gave written informed consent and, if able to judge, patients gave written informed consent too. The study was conducted in accordance with the Declaration of Helsinki and the Swiss law, which refers to the current Good Clinical Practice guidelines. It was approved by the respective local ethics committees (Ethikkommission Nord- und Zentralschweiz, Commission Cantonale d’Ethique de la Recherche sur l’être humain Genève (CCER), Commission Cantonale d’Ethique de la Recherche sur l’être humain Vaud (CER-VD), Ethikkommission Kinderkliniken Bern, Kantonale Ethikkommission Bern, Kantonale Ethikkommission Zürich; leading ethics committee: Kantonale Ethikkommission Bern) and had been registered at ClinicalTrials.gov (NCT02324231) before patient recruitment.

The SPOG 2015 FN Definition study primarily aimed to determine the safety of a higher (39.0°C) versus lower (38.5°C) fever limit using a non-blinded cluster-randomized controlled non-inferiority design. The 39.0°C fever limit has been found to be both safe and efficacious when compared to 38.5°C [10] and the risk to develop FN during chemotherapy has been described elsewhere [5]. Here, an analysis of observational outcomes predefined in the study protocol [12] is reported. The sample size calculation of the study protocol had not been done for this analysis, but for the primary study aim, details have been published [9].

### Patient selection and definitions

Patients treated with chemotherapy for cancer were consecutively screened. Inclusion criteria were age ≥ 12 months and < 18 years, diagnosis of any malignancy, and treatment with myelosuppressive chemotherapy expected to last ≥ 2 months, or ≥ 1 cycle of myeloablative chemotherapy followed by autologous hematopoietic stem cell transplantation. Allogenic stem cell transplantation patients were not included in this study.

Neutropenia was defined as an absolute neutrophil count < 0.5 G/L or < 1.0 G/L and expected to decline to < 0.5 G/L within 48 hours. FN was diagnosed at tympanic temperatures reaching the current fever limit (38.5°C or 39.0°C), but diagnosis below this limit (temperature ≥38·0°C; or ≥37·5°C in patients repeatedly receiving antipyretics) was allowed, if clinically indicated.

The three main outcomes were bacteremia, SMC, or SRE. Bacteremia was defined by detection of a recognized pathogen from ≥ 1 blood culture set(s) or common commensals from ≥ 2 blood culture sets drawn on separate occasions [10]. SMC was defined as death due to any cause during FN, admission to an ICU for organ support, or severe sepsis (including septic shock) according to established definitions [13]. SRE was defined as a composite outcome of bacteremia and/or SMC [10].

### Identification of published CDRs for external validation

**Fig 1:**
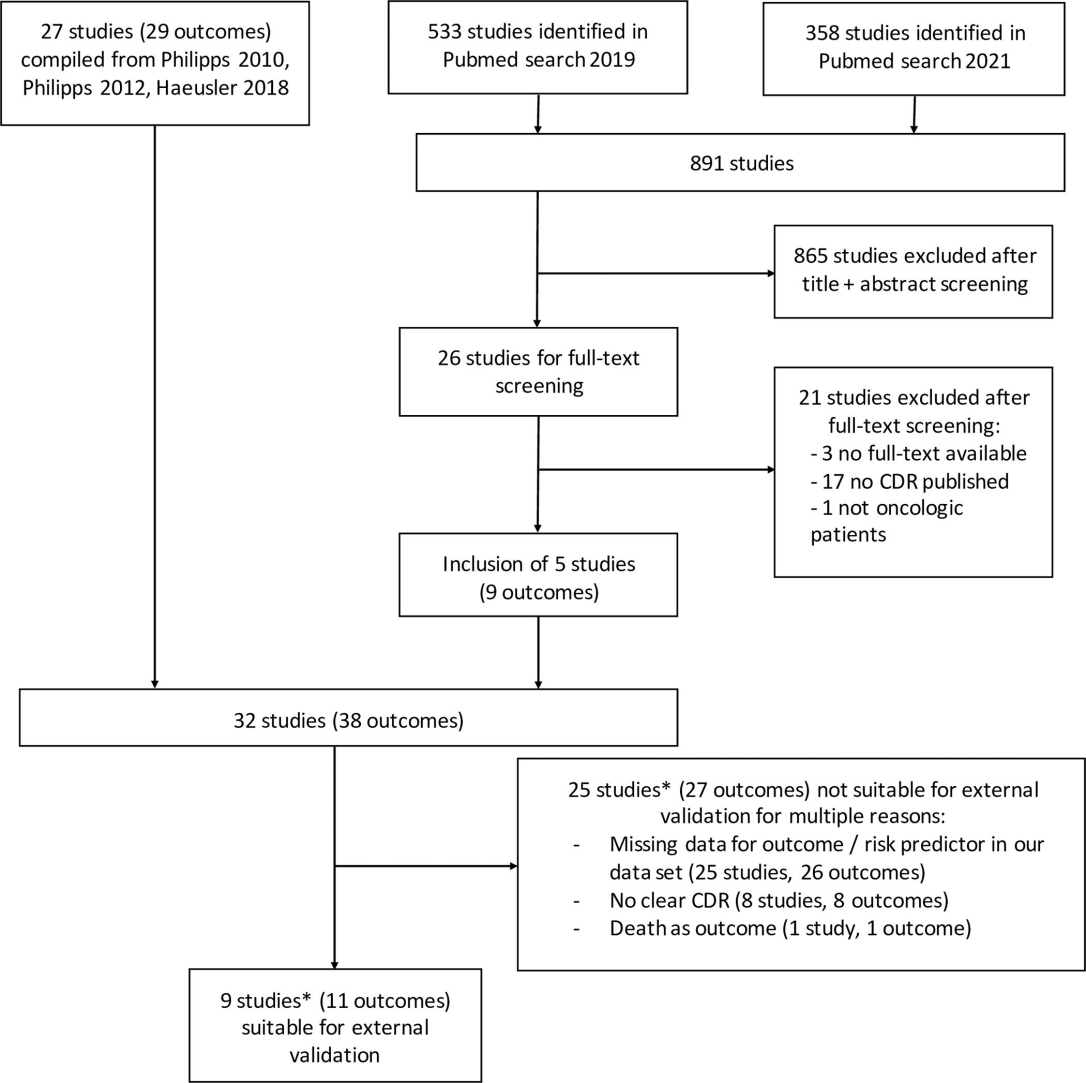
Flowchart literature search. * 2 studies with suitable and non-suitable outcomes.

A total of 27 CDR studies were compiled from two precedent systematic reviews [6, 7] and an external validation study [8]. To identify additional, newer studies, two PubMed searches for relevant pediatric CDRs published since 01/2016 were conducted on October 17, 2019 and on May 22, 2021, respectively. Using the search terms (fever OR febrile OR sepsis) AND (neutropenia OR neutropenic) AND (child OR children OR pediatric OR paediatric), 891 studies were identified. After screening through titles and abstracts 865 studies were excluded, thus 26 studies remained for full-text screening. Five studies [14–18] could then be added for further investigation to the 27 compiled in the three sources mentioned above [6–8] (Fig 1). For external validation, similar in-/exclusion criteria in the validation studies as in our original study, as well as availability (or computability) of described characteristics for CDRs and outcome were required. Finally, 9 studies with 11 CDRs could be used for external validation with our data set (Fig 1) [17–25].

### Statistical analysis

#### Development and internal validation of CDR

First, univariable associations of 15 clinical and five hematological characteristics with the three outcomes, i.e., bacteremia, SMC, and SRE; were analyzed using generalized linear mixed models, specifically three-level mixed logistic regression, with random intercepts per patient nested within centers. Interaction terms of clinical characteristics with the randomized temperature limit were non-significant in all these univariable analyses, thus confounding of this risk analysis by the randomized study design could be excluded. The following 15 clinical characteristics were analyzed: sex, age group at screening, type of malignancy, relapse status, chemotherapy intensity, presence of any central venous access device, bone marrow involvement, time since diagnosis, prior episodes of FN, prior episodes of FN with bacteremia, season (spring/summer vs autumn/winter), time of presentation (office vs out of office times), temperature at presentation ≥ 39°C, severely reduced general condition (i.e. a general deterioration in physical health, as judged by the treating physician) and systemic inflammatory response syndrome at presentation. Hematological characteristics assessed were: hemoglobin, leucocyte count, absolute neutrophil count, absolute monocyte count and platelet count at presentation.

Then, characteristics significantly associated in univariable analyses were used to construct multivariable prediction models (stepwise forward procedure, p < 0.05 for entry) [26]. To detect overfitting at each step, 100 random replications of 10-fold internal cross-validation were calculated. Overfitting was defined as decreasing median area under the receiver operating curve (AUROC) after inclusion of a new characteristic. No overfitting was detected in any of the three models.

Finally, risk prediction scores for all three outcomes were derived from the multivariable models. Their weights equaled the model coefficient multiplied by two, in order to increase discrimination between coefficients, and then rounded to the next integer. Negative weights were avoided by adding the lowest integer to all weights. The sums of these weights were the scores. For the corresponding CDRs, the thresholds of the scores were set to reach a sensitivity ≥ 90%.

For each of the three CDRs, 1000 random replications of 10-fold internal cross-validation were performed to estimate medians and 95% confidence intervals (CI) of parameters of predictive performance (sensitivity, specificity, negative and positive predictive values). Internal cross-validation included multivariable modeling with the given set of characteristics, derivation of the score and determination of the score threshold to reach the predefined sensitivity of 90%.

#### External validation of published CDRs

Parameters of predictive performances for the 11 published CDRs were calculated in our dataset (validation dataset, VD). Additionally, inclusion and exclusion criteria of the respective publication were applied to our data, resulting in restricted validation datasets (rVD). Parameters of predictive performance found in the derivation datasets (DD) were recalculated from published information. For CDRs stratifying patients in more than two groups [19, 20], only low versus intermediate/high risk were used to calculate parameters of predictive performances, but all risk groups were used to calculate AUROC. The SPOG-rule [24] was originally determined by predictors known at day two. For external validation, predictors known at FN presentation were used.

A CDR was considered reproducible if there was no significant difference in either sensitivity or specificity between the DD versus VD or DD versus rVD [9].

Three-level mixed logistic regression, with random intercepts per patient nested within centers, was used for model development, for internal cross-validation, and for external validation. Specifically, the glmer-function from the lme4-package [27] was used for mixed logistic regression, and the prop.test function for calculation of proportions with their 95% CIs. All tests were two-tailed and P-values <0.05 were considered statistically significant, except for interaction analyses, where P-values <0.01 were considered significant to account for the large number of interaction analyses done. All analyses were performed using R 4.0.0 [28].

## Results

### Patients and FN episodes

A total of 360 FN episodes in 158 patients (median age at screening 6 years, interquartile range 3 to 11) were reported in the study (S1 Fig, S1 Table). All episodes were analyzed here (median, 2 episodes per patient; interquartile range, 1 to 3 episodes, maximum 6 episodes). See S1 and S2 Tables for further demographic characteristics of patients and FN episodes.

Overall, an SRE was documented in 72 (20%) episodes. Of these, bacteremia was reported in 56 (16%) and an SMC in 30 (8%) episodes. Specifically, severe sepsis was described in 22 (6%) patients, and ICU admission in 16 (4%) patients. There were no deaths attributed to infection.

### CDRs to predict bacteremia, SMC or SRE

Nine out of 20 characteristics were significantly associated with bacteremia in univariable analysis (S3 Table). Four of them remained significantly and independently associated with bacteremia in multivariable analysis (Table 1). These were severely reduced general condition, leucocyte count <0.3 G/l, bone marrow involvement and type of malignancy. The predefined sensitivity of ≥ 90% was reached with a threshold set at ≥ 5 of 17 points for the corresponding CDR for bacteremia. At this threshold, the sensitivity was 96% (54 of 56) with a specificity of 32% (97 of 304) (Table 2).

**Table 1:** Association of characteristics with bacteremia, serious medical complications and safety relevant events, multivariable analysis and corresponding scores.

Outcomes	Bacteremia				Serious medical complications				Safety relevant events			
	Multivariable mixed logistic regression			Score	Multivariable mixed logistic regression			Score	Multivariable mixed logistic regression			Score
Characteristics	Coefficient	OR (95% CI)	p-value	max 17	Coefficient	OR (95% CI)	p-value	max 6	Coefficient	OR (95% CI)	p-value	max 15
Severely reduced general condition	1.56	4.75 (2.16–10.46)	<0.001	3	1.90	6.69 (2.71–16.49)	<0.001	4	1.84	6.27 (2.86–13.77)	<0.001	4
Bone marrow involvement	1.41	4.09 (1.43–11.71)	0.009	3	-	-	-	-	1.23	3.42 (1.09–10.73)	0.04	2
Leucocyte count <0.3 G/l	1.69	5.4 (2.69–10.86)	<0.001	3	-	-	-	-	1.54	4.64 (2.28–9.46)	<0.001	3
Thrombocyte count < 50 G/l	-	-	-	-	1.11	3.02 (1.16–7.87)	0.02	2	-	-	-	-
Type of malignancy Acute lymphoblastic leukemia	0	Reference	-	5	-	-	-	-	0	Reference	-	4
Acute myeloid leukemia	1.27	3.58 (0.98–13.02)	0.05	8	-	-	-	-	0.85	2.35 (0.55–10.05)	0.25	6
Hodgkin lymphoma	-0.30	0.74 (0.08–6.78)	0.79	4	-	-	-	-	-0.89	0.41 (0.04–4.34)	0.46	2
Non-Hodgkin lymphoma	-0.56	0.57 (0.21–1.58)	0.28	4	-	-	-	-	-0.74	0.48 (0.17–1.35)	0.16	3
CNS tumor	-2.58	0.08 (0.01–0.61)	0.02	0	-	-	-	-	-1.85	0.16 (0.04–0.66)	0.01	0
Other solid tumor	-1.52	0.22 (0.08–0.6)	0.003	2	-	-	-	-	-1.60	0.2 (0.07–0.55)	0.002	1

NOTE. Displayed are results of multivariable three-level mixed logistic regression. The score weights are coefficients multiplied by 2 and then rounded to the next integer. In score weights for type of malignancy, negative weights (range, -1 to -4) were avoided by adding 5 (for bacteremia) or 4 (for safety relevant events) to all weights.

Abbreviations: CNS, central nervous system, CI, confidence interval; OR, Odds ratio.

**Table 2:** Non cross-validated performance of new clinical decision rules (CDRs) in 360 fever in neutropenia (FN) episodes.

CDR	TP	TN	FP	FN	Outcome (%)	LR (%)	Sensitivity (95% CI)	Specificity (95% CI)	PPV (95% CI)	NPV (95% CI)
Bacteremia, AUROC 0.813										
• Threshold ≥ 3	56	54	250	0	56 (15.6)	54 (15)	100 (92–100)	17.8 (13.7–22.6)	18.3 (14.2–23.2)	100 (91.7–100)
• Threshold ≥ 4	56	75	229	0	56 (15.6)	75 (20.8)	100 (92–100)	24.7 (20–30)	19.6 (15.3–24.8)	100 (93.9–100)
• Threshold ≥ 5*	**54**	**97**	**207**	**2**	**56 (15.6)**	**99 (27.5)**	**96.4 (86.6–99.4)**	**31.9 (26.8–37.5)**	**20.7 (16–26.2)**	**98 (92.2–99.6)**
• Threshold ≥ 6	42	226	78	14	56 (15.6)	240 (66.7)	75 (61.4–85.2)	74.3 (69–79.1)	35 (26.7–44.3)	94.2 (90.2–96.7)
SMC, AUROC 0.690										
• Threshold ≥ 1	28	149	181	2	30 (8.3)	151 (41.9)	93.3 (76.5–98.8)	45.2 (39.7–50.7)	13.4 (9.2–18.9)	98.7 (94.8–99.8)
• Threshold ≥ 2*	**28**	**149**	**181**	**2**	**30 (8.3)**	**151 (41.9)**	**93.3 (76.5–98.8)**	**45.2 (39.7–50.7)**	**13.4 (9.2–18.9)**	**98.7 (94.8–99.8)**
• Threshold ≥ 3	14	292	38	16	30 (8.3)	308 (85.6)	46.7 (28.8–65.4)	88.5 (84.4–91.6)	26.9 (16–41.3)	94.8 (91.5–96.9)
SRE, AUROC 0.799										
• Threshold ≥ 2	71	53	235	1	72 (20)	54 (15)	98.6 (91.5–99.9)	18.4 (14.2–23.5)	23.2 (18.7–28.4)	98.1 (88.8–99.9)
• Threshold ≥ 3	71	58	230	1	72 (20)	59 (16.4)	98.6 (91.5–99.9)	20.1 (15.8–25.3)	23.6 (19–28.9)	98.3 (89.7–99.9)
• Threshold ≥ 4*	**67**	**93**	**195**	**5**	**72 (20)**	**98 (27.2)**	**93.1 (83.7–97.4)**	**32.3 (27–38.1)**	**25.6 (20.5–31.4)**	**94.9 (87.9–98.1)**
• Threshold ≥ 5	53	217	71	19	72 (20)	236 (65.6)	73.6 (61.7–83)	75.3 (69.9–80.1)	42.7 (34–51.9)	91.9 (87.5–95)
• Threshold ≥ 6	50	220	68	22	72 (20)	242 (67.2)	69.4 (57.3–79.5)	76.4 (71–81.1)	42.4 (33.4–51.8)	91.9 (86.4–94.1)

* Threshold at the predefined sensitivity of ≥90%

Abbreviations: AUROC, area under the receiver operating curve; CDR, clinical decision rule; CI, confidence interval; FN, false negative; FP, false positive; LR, low risk; NPV, negative predictive value; PPV, positive predictive value; TN, true negative; TP, true positive.

Three out of 20 characteristics were significantly associated with SMC in univariable analysis (S3 Table). Two of them remained significantly and independently associated with SMC in the multivariable model (Table 1). These were severely reduced general condition and platelet count <50 G/l. The predefined sensitivity of ≥ 90% was reached with a threshold set at ≥ 2 out of 6 points for the corresponding CDR for SMC. At this threshold, the sensitivity was 93% (28 of 30) with a specificity of 45% (149 of 330) (Table 2).

Six out of 20 characteristics were significantly associated with SRE in univariable analysis (S3 Table). Four of them remained significantly and independently associated with SRE in the multivariable model (Table 1). These were severely reduced general condition, leucocyte count <0.3 G/l, bone marrow involvement and type of malignancy. The predefined sensitivity of ≥ 90% was reached with a threshold set at, ≥ 4 out of 15 points for the corresponding CDR for SRE. At this threshold, the sensitivity was 93% (67 of 72) with a specificity of 32% (93 of 288) (Table 2).

### Internal cross-validation

As expected, median cross-validated sensitivities and specificities were slightly lower than non-cross-validated sensitivities and specificities for the prediction of bacteremia, SMC, or SRE. Nevertheless, none of the differences were significant (S4 Table).

### External validation of published CDRs

External validation on 11 CDRs from 9 different studies was performed, using the entire dataset of 360 episodes of FN (VD), and the respective rVDs. Four of these CDRs predicted bacteremia, one CDR each predicted serious bacterial infection, likely bacterial infection, invasive bacterial infection, severe infection complications, adverse events, severe adverse outcomes, and ICU admission (Table 3).

**Table 3:** Overview on externally validated clinical decision rules (CDRs): Inclusion and exclusion criteria, characteristics and outcomes.

CDR	Inclusion criteria	Exclusion criteria	High risk criteria	High risk outcome
Rackoff 1996*	Cancer or hematologic malignancy; fever ≥ 38.5°C once or ≥38.0°C 3× during a 24-h period*; ANC < 0.5 G/l; outpatient	Inpatient onset FN	High risk = AMC < 0.1 G/l and temperature ≥39°C Intermediate risk = AMC < 0.1 G/l and temperature < 39°C Low risk = AMC ≥ 0.1 G/l	**Bacteremia** (defined as a positive blood culture)
Klaassen 2000*	Cancer or hematologic malignancy, fever > 38.5°C once or >38.0°C during 12 hour period*; ANC ≤ 0.5 or between 0.5 and 1.0 G/l and expected to fall, outpatient	New diagnosis cancer, HSCT within 6 months, comorbidity on presentation including severe mucositis and pneumonia	AMC < 0.1 G/l	**Significant bacterial** **Infection—**defined as blood or urine culture positive for bacteria, interstitial or lobar consolidation on chest x-ray, or unexpected death from infection (patient not palliative)
Baorto 2001*	Cancer or hematologic malignancy, fever ≥ 38.0°C; ANC ≤ 0.5 G/l	Age < 1 y, previous HSCT	AMC < 0.155 G/l	**Bacteremia** (not defined)^†^
Madsen 2002*	Cancer or hematologic malignancy; fever ≥ 38.5°C once or ≥38.0°C 3× during a 24-h period*; ANC ≤ 0.5 G/l; outpatient	Inpatient onset of FN, HSCT, AML patients in intensive timing theraoy	Temperature > = 39.5°C and AMC ≤0.01G/l	**positive blood culture** ^†^
Rondinelli 2006*	Cancer or hematologic Malignancy; fever ≥ 38.1°C once or > 37.8°C on 3 separate occasions measured within a period of 24 hours*; ANC (segmented granulocytes and rods) < 0.5 G/l or 0.5–1.0 that tended to drop in 72 hours, outpatient, first episode of FN	HSCT (autolog and allogenic), not the first episode of FN; inpatient onset of FN	Score 2.5–5 = low risk Score 5.5–9 = intermediate risk Score ≥ 9 = high risk Age ≤5 y = 1, 2. CVAD = 2, Clinical site = 4.5, Fever >38.5°C = 1, Hemoglobin ≤ 70g/l = 1, upper respiratory tract infection = 2.5	**Severe infection complication** was defined as the presence of sepsis and/or shock and/or bacteremia or fungemia from blood sample, and/or death from an infectious process during a FN episode. The presence of any infectious agent in a blood sample was considered as bacteremia. Septicemia was defined as a syndrome of systemic inflammatory response (involving ≥ 2 of the following characteristics: tachycardia, tachypnea, hypothermia,or hyperthermia, with positive blood culture or clinical and laboratorial infection detected and adequate peripheral perfusion). Patients were considered in septic shock when severe sepsis was observed, with clinical signs of hypoperfusion and blood hypotension, who no longer answered to fluids and who needed inotropic doses to maintain hemodynamic balance.
SPOG-AE (Ammann) 2010*	Cancer or hematologic malignancy; fever ≥ 38.5°C once or ≥38.0°C during ≥2 hours*; ANC ≤ 0.5 G/l; outpatient	Myeloablative chemotherapy; AE known at presentation	Applied after 24 hours^‡^ Total score ≥ 9 = high risk of AE. Score for preceding chemotherapy more intensive than ALL maintenance = 4; Hb ≥ 90 g/l = 5; leukocyte count < 0.3 G/L = 3; platelet count < 50 G/L = 3	**Adverse outcome**, defined as a SMC (death, complication requiring ICU and potentially life-threatening complication as judged by the treating physician) as a result of infection, MDI (positive bacterial or fungal culture from a normally sterile site and detection of a viral antigen by PCR) and radiologically confirmed pneumonia. Bacteremia not defined^†^
Hakim 2010*	Cancer or hematologic malignancy; fever ≥ 38.3°C or ≥38.0°C for ≥1 hour*; ANC ≤ 0.5 G/l; outpatient	HSCT; inpatient onset FN	Total score ≥ 24 = high risk of invasive bacterial infection. Score for cancer diagnosis: AML = 20, ALL/lymphoma = 7, solids = 0 points; Clinical presentation serious unwell or toxic = 14 points; Fever ≥ 39°C at presentation = 11 points; ANC < 0.1 G/l = 10 points	**Proven invasive bacterial Infection**, defined as isolation of a pathogen from a sterile body site or as proven by histology. **Culturenegative Sepsis**, defined as a systemic response to a possible infection because of hemodynamic instability, focal or multiple organ involvement or altered mental status or lethargy. **Bacteremia** defined as a recognized pathogen cultured from one or more blood cultures or common commensals cultured from two or more blood cultures.
Suttitossatam 2020*	fever ≥ 38.3°C or ≥ 38.0°C persisting >1 hour*; ANC < 0.5 G/l or ANC < 1.0 G/l with a predicted decrease to < 0.5 G/l	Age < 1 year	Age ≥ 10 years	**Severe adverse outcomes** as hypotension (is determined by age and systolic blood pressure, in line with Pediatric Advanced Life Support (PALS) Guidelines) or shock; respiratory failure (the need for noninvasive respiratory support or mechanical ventilation); death
AUS (Haeusler) 2020*	Cancer or hematologic Malignancy; fever ≥ 38°C; ANC < 1.0 G/l; in- and outpatient	HSCT in last 3 months; treatment commenced at a non-participating site already receiving concurrent intravenous or oral antibiotics (excluding prophylaxis)	Score ≥ 1 = High risk preceding chemotherapy more intensive than ALL maintenance = 1; WCC < 0.3 G/l = 1; platelet <50 g/L = 1	Three outcomes were analyzed: • **Likely bacterial infectionI** (any infection with a microbiologically documented bacterial cause or that was clinically documented in categories typically attributed to bacterial infection, including pneumonia, skin and soft-tissue infection, osteomyelitis or myositis, enterocolitis, otitis media or externa, sinusitis, epididymoorchitis, centralvenous catheter pocket or tunnel infection, pharyngitis, perianal abscess or cellulitis, peritonitis or lymphadenitis) • **Bacteremia** (recognised pathogen (including organisms associated with mucosal barrier injury in the setting of mucositis or neutropenia) from ≥ 1 blood culture set or common commensals from ≥ 2 blood culture sets drawn on separate occasions) • **ICU-Admission**

* for external crossvalidation with restricted dataframe these definitions were modified for validation because of available data (inclusion of all events which reached the lower temperature limit once).

^**†**^ if not otherwise specified, international consensus definition was used for validation (Haeusler GM, 2015, Pediatr Blood Cancer).

^‡^ Application at FN presentation for external cross-validation.

Abbreviations: AE, adverse event; ALL, acute lymphoblastic leukemia; AML, acute myeloid leukemia; AMC, absolute monocyte count; ANC, absolute neutrophil count; FN, fever in neutropenia; HSCT, hematopoietic stem cell transplantation; ICU, intensive care unit; MDI, microbiological defined infection; PCR, polymerase chain reaction; SMC, serious medical complication, WCC, white cell count.

**Fig 2:**
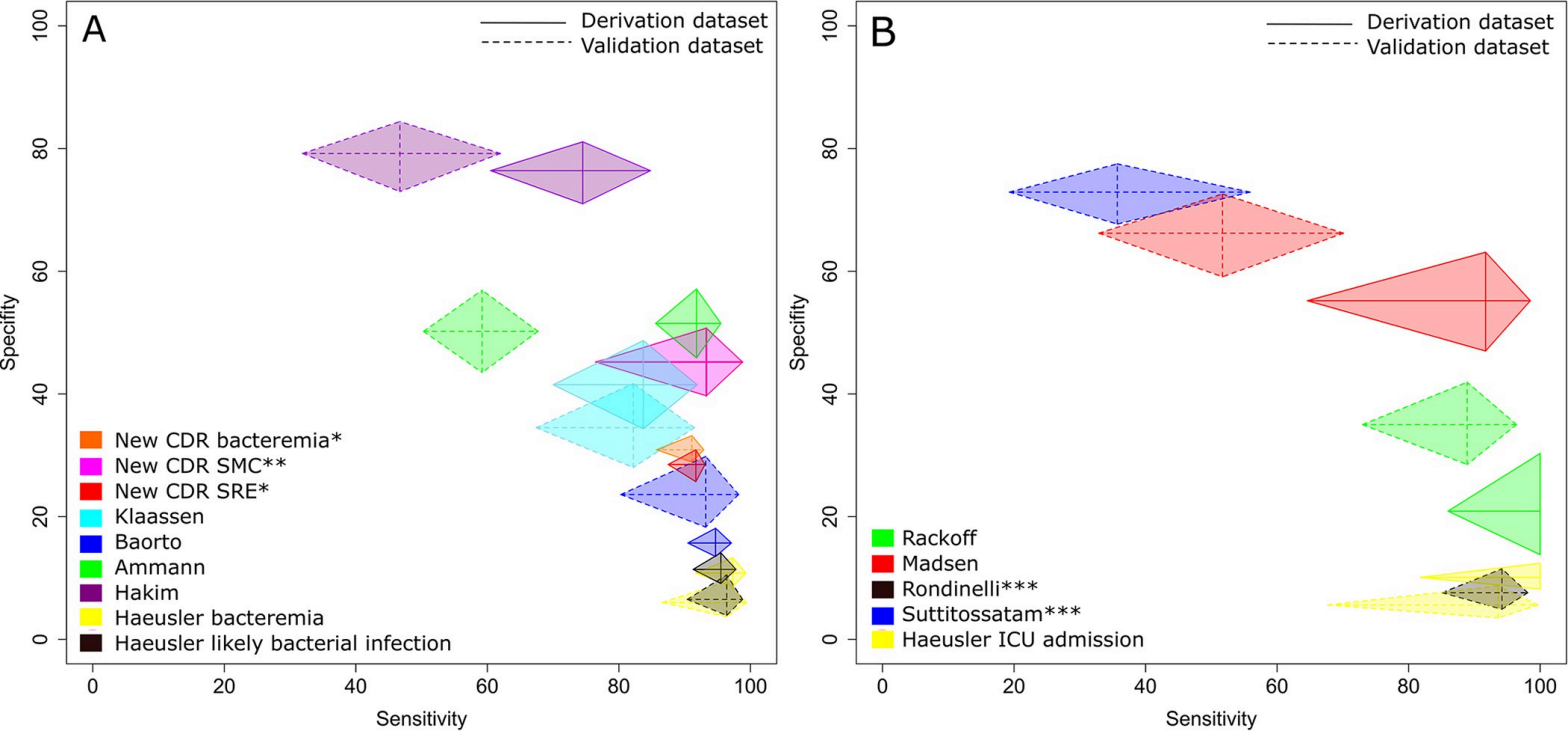
Sensitivity and specificity of internal crossvalidation of the new CDRs and external validation of published CDRs. A) New CDRs and published CDRs with reproducible sensitivity and/or specificity. B) Published CDRs with non-reproducible sensitivity and specificity or missing information. * Results of internal cross-validation. ** Non cross-validated results. ***Only results for validation dataset available due to missing information in the original publication.

Of the 11 CDRs analyzed, only eight could be assessed for reproducibility because of missing information in two of the original publications [17, 23] and use of a different cut-off. [19] Of the remaining eight CDRs, six showed reproducibility for sensitivity [18, 20, 21] or specificity [20, 24, 25], thus only one [20] for both sensitivity and specificity (Fig 2, S4 Table). All reproducibility results were identical for external cross-validation on both the VD and the rVD.

## Discussion

This analysis developed CDRs predicting the risk of bacteremia, SMC, or SRE in pediatric patients with cancer and FN. Bacteremia was observed in 15.6%, SMC in 8.3%, SRE in 20% of FN episodes. The CDRs for bacteremia and SRE used four easily accessible characteristics and the CDR for SMC only two characteristics.

For bacteremia, all patients with acute lymphoblastic or myeloid leukemia classified as high risk. In addition all patients, besides the ones with a central nervous system (CNS) malignancy, classified as high risk, when presenting with severely reduced general condition, leucocyte count <0.3G/l or bone marrow involvement. Neurological deterioration or other complications of cerebral surgical interventions may lead to a severely reduced general condition even without bacteremia.

For SMC, patients classified as high risk when presenting either with severely reduced general condition or thrombocyte count <50G/l. SMC alone is not a reasonable outcome for CDR implementation at presentation with FN, as it does not include patients with bacteremia, which do need in-hospital antibiotic therapy and should therefore not be excluded from prediction. However, prediction of a low risk for SMC may help to decide that treatment can be reduced when blood cultures remain negative.

For SRE, all patients with acute lymphoblastic or myeloid leukemia and all patients presenting with severely reduced general condition classified as high risk. Additional characteristics lead to a high-risk classification if present in combination (e.g. leucocyte count <0.3G/l, bone marrow involvement, type of malignancy).

Performance of all three scores, when evaluated by internal cross-validation, has comparable or better predictive values than published CDRs. The characteristics used for these new CDRs are among the dominant themes used in published CDRs e.g. underlying diagnosis, chemotherapeutic regimen, clinical and laboratory parameters [7]. Thus, these new CDRs are essentially in line with published CDRs and do not identify novel factors not previously described.

Further, this analysis gives an actualized overview and external validation of published CDRs. Several of them had been validated before, the newest two (Suttitossatam [17], Haeusler [18]) and Madsen [22] have, to our knowledge, not been externally validated until today. Many of the CDRs developed in the recent years include inflammatory parameters such as c-reactive protein [14, 29, 30], and procalcitonin [15, 31, 32], but also less accessible values as interleukin 6 [15, 32], and interleukin 8 [32, 33], that have not been obtained in our dataset. Several publications with even newer, more experimental parameters (citrulline, proadrenomedullin, presepsin a.o.) have been published, but none of them established a clear CDR [34–37].

Of the 11 published CDRs analyzed, 8 could be assessed here. Only Klaassen [20] showed to be reproducible with both overlapping specificity and sensitivity. Another five CDRs [18, 21, 24, 25] showed overlapping results in either sensitivity or specificity. The reproducibility of two published CDRs could not be assessed due to partially missing information in the original publication. [17, 23] One published CDR [19] compared low/intermediate versus high risk, therefore a direct comparison of the validation parameters was not possible. As described in previous analyses [7], poor results in validation may be due to geographical variations. The reason for a much lower sensitivity in the validation of the CDR from Ammann et al. [24], that was developed in the same country and thus in a very similar population, may be due to the different time point of prediction (at reassessment after 8 to 24 hours of inpatient therapy).

In the latest years, several literature reviews and external validations have been published by Haeusler et al. from Australia. [8, 9, 18, 38] Geographically and climatically different but with a similar socioeconomic situation, our results have several analogies concerning the reproducibility of the CDRs. As already demonstrated by Haeusler 2018 [8], we could not find any statistically relevant differences between the restricted and non-restricted validation dataset, and the restriction could potentially be omitted in future validation studies.

The main limitation of this analysis stems from the fact that the underlying study randomized two different temperature limits defining fever. However, relevant confounding of the analyses presented here could be excluded by non-significant results in the corresponding univariable interaction analyses. Other limitations are the single-country setting and that further laboratory markers were not accessible, which precluded external validation of a large number of published CDRs.

Major strengths of this analysis are that it is based on data from a prospective multicenter study; on patients with a wide spectrum of malignancies, and that the scores rely on few routinely available clinical and laboratory characteristics. Internal cross-validation avoided overfitting of the multivariable models and consequently the score.

In conclusion, this analysis developed CDRs predicting bacteremia, SMC, and SRE in children with cancer presenting with FN. They are essentially in line with published CDRs and do not identify novel factors not previously described. In addition, external validation of 8 published CDRs identified 5 of them to show reproducible results for either sensitivity or for specificity in our setting. Only one [20] showed reproducible results for both sensitivity and specificity, but with poor absolute indices of predictive performance (AUC ROC, specificity at predefined sensitivities). Which of these six CDRs should be chosen by a specific pediatric oncology department for clinical implementation will mainly depend on the local weighing of sensitivity versus specificity, and the comparability of clinical settings. Validation of published CDRs in a variety of pediatric oncology populations is fundamental to find the best balance between sensitivity and specificity and will help to further improve management of FN.

## Supporting information

S1 Fig Flow chart of patients included. Abbreviations: FN, fever in neutropenia, HSCT, haematopoietic stem cell transplantation; IC, informed consent.
(PDF)

S1 Table Characteristics of the 158 patients with fever in neutropenia (FN) studied. (PDF)

S2 Table Characteristics of the 360 fever in neutropenia (FN) episodes studied. NOTE: if not otherwise indicated characteristics were known in all 360 episodes.
(PDF)

S3 Table Association of characteristics with bacteremia, serious medical complications and safety relevant events, univariable analysis. *result of two level mixed regression due to model failure of the three-level mixed model. Abbreviations: CI, confidence interval; OR, Odds ratio; FN, fever in neutropenia; FN-BACT, fever in neutropenia with bacteremia.
(PDF)

S4 Table Results of internal crossvalidation of the new CDRs and external validation of published CDRs. Abbreviations: CDR, clinical decision rule; CI, confidence interval; DD, derivation dataset; ICU, intensive care unit; LR, low risk; NPV, negative predictive value; PPV, positive predictive value; TN, true negative; TP, true positive; VD, validation dataset; rVD, restricted validation dataset. Bold = reproducibility criteria fulfilled for sensitivity / specificity.
(PDF)

## References

[1] Phillips B, Depani S, Morgan J. What do families want to improve in the management of paediatric febrile neutropenia during anti-cancer treatment? Report of a patient/public involvement group. BMJ Paediatr Open 2019, 3(1):e000398. doi: 10.1136/bmjpo-2018-000398 30957027 PMC6422240

[2] Morgan JE, Cleminson J, Atkin K, Stewart LA, Phillips RS. Systematic review of reduced therapy regimens for children with low risk febrile neutropenia. Support Care Cancer 2016, 24(6):2651–2660. doi: 10.1007/s00520-016-3074-9 26757936

[3] Brack E, Bodmer N, Simon A, Leibundgut K, Kuhne T, Niggli FK, et al. First-day step-down to oral outpatient treatment versus continued standard treatment in children with cancer and low-risk fever in neutropenia. A randomized controlled trial within the multicenter SPOG 2003 FN study. Pediatr Blood Cancer 2012, 59(3):423–430. 22271702 10.1002/pbc.24076

[4] Lehrnbecher T, Robinson P, Ammann RA, Fisher B, Patel P, Phillips R, et al. Guideline for the Management of Fever and Neutropenia in Pediatric Patients With Cancer and Hematopoietic Cell Transplantation Recipients: 2023 Update. J Clin Oncol 2023, 20;41(9):1774–1785. doi: 10.1200/JCO.22.02224 36689694 PMC10022858

[5] Lavieri L, Koenig C, Bodmer N, Agyeman PKA, Scheinemann K, Ansari M, et al. Predicting fever in neutropenia with safety-relevant events in children undergoing chemotherapy for cancer: The prospective multicenter SPOG 2015 FN Definition Study. Pediatr Blood Cancer 2021, 68(12):e29253. doi: 10.1002/pbc.29253 34310027

[6] Phillips B, Wade R, Stewart LA, Sutton AJ. Systematic review and meta-analysis of the discriminatory performance of risk prediction rules in febrile neutropaenic episodes in children and young people. Eur J Cancer 2010, 46(16):2950–2964. doi: 10.1016/j.ejca.2010.05.024 20621468 PMC2981857

[7] Phillips RS, Lehrnbecher T, Alexander S, Sung L. Updated systematic review and meta-analysis of the performance of risk prediction rules in children and young people with febrile neutropenia. PLoS One 2012, 7(5):e38300. doi: 10.1371/journal.pone.0038300 22693615 PMC3365042

[8] Haeusler GM, Thursky KA, Slavin MA, Mechinaud F, Babl FE, Bryant P, et al. External Validation of Six Pediatric Fever and Neutropenia Clinical Decision Rules. Pediatr Infect Dis J 2018, 37(4):329–335. doi: 10.1097/INF.0000000000001777 28877157

[9] Haeusler GM, Thursky KA, Slavin MA, Babl FE, De Abreu Lourenco R, Allaway Z, et al. Risk stratification in children with cancer and febrile neutropenia: A national, prospective, multicentre validation of nine clinical decision rules. EClinicalMedicine 2020, 18:100220. doi: 10.1016/j.eclinm.2019.11.013 31993576 PMC6978200

[10] Koenig C, Bodmer N, Agyeman PKA, Niggli F, Adam C, Ansari M, et al. 39.0 degrees C versus 38.5 degrees C ear temperature as fever limit in children with neutropenia undergoing chemotherapy for cancer: a multicentre, cluster-randomised, multiple-crossover, non-inferiority trial. The Lancet Child & adolescent health 2020, 4(7):495–502. 32497520 10.1016/S2352-4642(20)30092-4

[11] Harris PA, Taylor R, Thielke R, Payne J, Gonzalez N, Conde JG. Research electronic data capture (REDCap)—a metadata-driven methodology and workflow process for providing translational research informatics support. Journal of biomedical informatics 2009, 42(2):377–381. doi: 10.1016/j.jbi.2008.08.010 18929686 PMC2700030

[12] Swiss Paediatric Oncology Group. SPOG 2015 FN Definition Protocol 2016. 2016 November 23 [cited 2023 Mai 5] In: SPOG Homepage [Internet]. Available from: https://www.spog.ch/wp-content/uploads/2020/03/SPOG_FN_Protocol1.1_20161123_PDF.pdf.

[13] Goldstein B, Giroir B, Randolph A. International Consensus Conference on Pediatric S: International pediatric sepsis consensus conference: definitions for sepsis and organ dysfunction in pediatrics. Pediatric critical care medicine: a journal of the Society of Critical Care Medicine and the World Federation of Pediatric Intensive and Critical Care Societies 2005, 6(1):2–8. 15636651 10.1097/01.PCC.0000149131.72248.E6

[14] Oberoi S, Das A, Trehan A, Ray P, Bansal D. Can complications in febrile neutropenia be predicted? Report from a developing country. Support Care Cancer 2017, 25(11):3523–3528. doi: 10.1007/s00520-017-3776-7 28601903

[15] van der Galien HT, Loeffen EAH, Miedema KGE, Tissing WJE. Predictive value of PCT and IL-6 for bacterial infection in children with cancer and febrile neutropenia. Support Care Cancer 2018, 26(11):3819–3826. doi: 10.1007/s00520-018-4249-3 29777383 PMC6182367

[16] Segulja S, Ruzic A, Dujmic D, Bazdaric K, Roganovic J. Simple predictors of the re- occurrence of severe febrile neutropenia episode: a single-center retrospective cohort study in pediatric patients with malignant diseases. Croat Med J 2019, 60(1):20–25. doi: 10.3325/cmj.2019.60.20 30825274 PMC6406065

[17] Suttitossatam I, Satayasai W, Sinlapamongkolkul P, Pusongchai T, Sritipsukho P, Surapolchai P. Predictors of severe adverse outcomes in febrile neutropenia of pediatric oncology patients at a single institute in Thailand. Pediatr Hematol Oncol 2020, 37(7):561–572. doi: 10.1080/08880018.2020.1767243 32543327

[18] Haeusler GM, Phillips R, Slavin MA, Babl FE, De Abreu Lourenco R, Mechinaud F, et al. Re-evaluating and recalibrating predictors of bacterial infection in children with cancer and febrile neutropenia. EClinicalMedicine 2020, 23:100394. doi: 10.1016/j.eclinm.2020.100394 32637894 PMC7329706

[19] Rackoff WR, Gonin R, Robinson C, Kreissman SG, Breitfeld PB. Predicting the risk of bacteremia in childen with fever and neutropenia. J Clin Oncol 1996, 14(3):919–924. doi: 10.1200/JCO.1996.14.3.919 8622040

[20] Klaassen RJ, Goodman TR, Pham B, Doyle JJ. "Low-risk" prediction r38ule for pediatric oncology patients presenting with fever and neutropenia. J Clin Oncol 2000, 18(5):1012–1019. 10694551 10.1200/JCO.2000.18.5.1012

[21] Baorto EP, Aquino VM, Mullen CA, Buchanan GR, DeBaun MR. Clinical parameters associated with low bacteremia risk in 1100 pediatric oncology patients with fever and neutropenia. Cancer 2001, 92(4):909–913. doi: 10.1002/1097-0142(20010815)92:4&lt;909::aid-cncr1400&gt;3.0.co;2-h 11550165

[22] Madsen K, Rosenman M, Hui S, Breitfeld PP. Value of electronic data for model validation and refinement: bacteremia risk in children with fever and neutropenia. J Pediatr Hematol Oncol 2002, 24(4):256–262. doi: 10.1097/00043426-200205000-00008 11972092

[23] Rondinelli PI, Ribeiro Kde C, de Camargo B. A proposed score for predicting severe infection complications in children with chemotherapy-induced febrile neutropenia. J Pediatr Hematol Oncol 2006, 28(10):665–670. doi: 10.1097/01.mph.0000212996.94929.0b 17023827

[24] Ammann RA, Bodmer N, Hirt A, Niggli FK, Nadal D, Simon A, et al. Predicting adverse events in children with fever and chemotherapy-induced neutropenia: the prospective multicenter SPOG 2003 FN study. J Clin Oncol 2010, 28(12):2008–2014. doi: 10.1200/JCO.2009.25.8988 20231680

[25] Hakim H, Flynn PM, Srivastava DK, Knapp KM, Li C, Okuma J, et al. Risk prediction in pediatric cancer patients with fever and neutropenia. Pediatr Infect Dis J 2010, 29(1):53–59. doi: 10.1097/INF.0b013e3181c3f6f0 19996816

[26] Altman DG. Practical Statistics for Medical Research. London, United Kingdom: Chapman & Hall; 1991.

[27] D Bates MM B Bolker, S Walker Fitting Linear Mixed-Effects Models Using lme4. J Stat Softw 2015:67:64

[28] R Core Team. R: A language and environment for statistical computing. Vienna, Austria: R Foundation for Statistical Computing; 2014.

[29] Ammann RA, Hirt A, Luthy AR, Aebi C. Identification of children presenting with fever in chemotherapy-induced neutropenia at low risk for severe bacterial infection. Med Pediatr Oncol 2003, 41(5):436–443. doi: 10.1002/mpo.10320 14515382

[30] Santolaya ME, Alvarez AM, Becker A, Cofre J, Enriquez N, O’Ryan M, et al. Prospective, multicenter evaluation of risk factors associated with invasive bacterial infection in children with cancer, neutropenia, and fever. J Clin Oncol 2001, 19(14):3415–3421. doi: 10.1200/JCO.2001.19.14.3415 11454890

[31] Hemming V, Jakes AD, Shenton G, Phillips B. Prospective cohort study of procalcitonin levels in children with cancer presenting with febrile neutropenia. BMC Pediatr 2017, 17(1):2. doi: 10.1186/s12887-016-0766-8 28056911 PMC5217195

[32] Srinivasan A, Kumar N, Scott JX. Evaluation of serum procalcitonin, serum interleukin-6, and interleukin-8 as predictors of serious infection in children with febrile neutropenia and cancer. Indian J Cancer 2021, 58(2):185–189. doi: 10.4103/ijc.IJC_808_18 33402576

[33] Miedema KG, Tissing WJ, Abbink FC, Ball LM, Michiels EM, van Vliet MJ, et al. Risk-adapted approach for fever and neutropenia in paediatric cancer patients—a national multicentre study. Eur J Cancer 2016, 53:16–24. doi: 10.1016/j.ejca.2015.10.065 26700076

[34] De Pietri S, Frandsen TL, Christensen M, Grell K, Rathe M, Muller K. Citrulline as a biomarker of bacteraemia during induction treatment for childhood acute lymphoblastic leukaemia. Pediatr Blood Cancer 2021, 68(1):e28793. doi: 10.1002/pbc.28793 33155402

[35] Arikan K, Karadag-Oncel E, Aytac S, Cetin M, Cengiz AB, Gumruk F, et al. Usage of Plasma Presepsin, C-Reactive Protein, Procalcitonin and Proadrenomedullin to Predict Bacteremia in Febril Neutropenia of Pediatric Hematological Malignancy Patients. Lab Med 2021, 52(5):477–484. doi: 10.1093/labmed/lmab002 33851202

[36] Kesik V, Atas E, Gulcan KY, Aydin FN, Babacan O, Gulgun M, et al. Adrenomedullin predicts high risk and culture positivity in children with solid tumors suffering from neutropenic fever. J Infect Chemother 2016, 22(9):617–621. doi: 10.1016/j.jiac.2016.06.007 27400951

[37] Baraka A, Zakaria M. Presepsin as a diagnostic marker of bacterial infections in febrile neutropenic pediatric patients with hematological malignancies. Int J Hematol 2018, 108(2):184–191. doi: 10.1007/s12185-018-2447-x 29616457

[38] Haeusler GM, Thursky KA, Mechinaud F, Babl FE, De Abreu Lourenco R, Slavin MA, et al. Predicting Infectious ComplicatioNs in Children with Cancer: an external validation study. Br J Cancer 2017, 117(2):171–178. doi: 10.1038/bjc.2017.154 28609435 PMC5520507

