# Supplementary material for: Outcome prediction in pediatric fever in neutropenia: Development of clinical decision rules and external validation of published rules based on data from the prospective multicenter SPOG 2015 FN definition study
Source: PLoS One. 2023 Aug 2;18(8):e0287233. doi: 10.1371/journal.pone.0287233 (PMC10395874; doi:10.1371/journal.pone.0287233)
Supplement: S1 Fig — Abbreviations: FN, fever in neutropenia, HSCT, haematopoietic stem cell transplantation; IC, informed consent. (PDF) [file pone.0287233.s001.pdf]

S1 Figure Flow chart of patients included

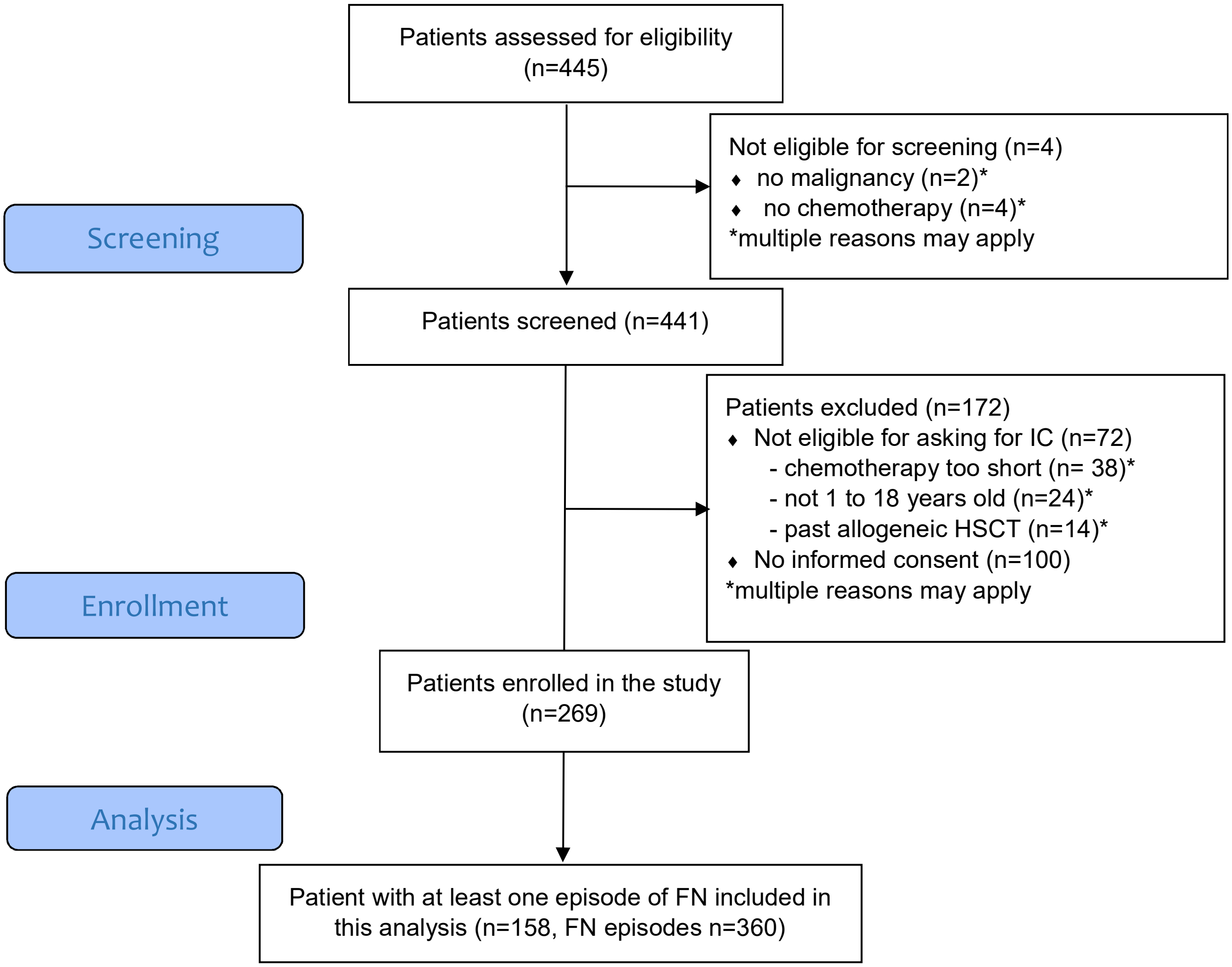

Abbreviations: FN, fever in neutropenia, HSCT, haematopoietic stem cell transplantation; IC, informed consent
