# Supplementary material for: Outcome prediction in pediatric fever in neutropenia: Development of clinical decision rules and external validation of published rules based on data from the prospective multicenter SPOG 2015 FN definition study
Source: PLoS One. 2023 Aug 2;18(8):e0287233. doi: 10.1371/journal.pone.0287233 (PMC10395874; doi:10.1371/journal.pone.0287233)
Supplement: S1 Table — (PDF) [file pone.0287233.s002.pdf]

S1 Table Characteristics of the 158 patients with fever in neutropenia (FN) studied

Patient characteristics	n (%)
Gender	
- Female	89 (56%)
- Male	69 (44%)
Malignancy related characteristics	
Type of malignancy	
- Acute lymphoblastic leukemia	70 (44%)
- Acute myeloid leukemia	6 (4%)
- Hodgkin lymphoma	6 (4%)
- Non-Hodgkin lymphoma	18 (11%)
- Central nervous system tumor	16 (10%)
- Other solid tumor	42 (27%)
Relapsed malignancy	9 (6%)
Bone marrow involvement	17 (11%)
Therapy related characteristics	
Chemotherapy intensity	
- 1	12 (8%)
- 2	124 (78%)
- 3	13 (8%)
- 4	9 (6%)
Central venous access device	157 (99%)
Outcomes	
- Bacteremia	26 (16%)
- Serious medical complication	12 (8%)
- Safety relevant event	31 (20%)
- Severe sepsis	9 (6%)
- Intensive care unit admission	8 (5%)
- Death	0 (0%)
