# Supplementary material for: Outcome prediction in pediatric fever in neutropenia: Development of clinical decision rules and external validation of published rules based on data from the prospective multicenter SPOG 2015 FN definition study
Source: PLoS One. 2023 Aug 2;18(8):e0287233. doi: 10.1371/journal.pone.0287233 (PMC10395874; doi:10.1371/journal.pone.0287233)
Supplement: S2 Table — NOTE: if not otherwise indicated characteristics were known in all 360 episodes. (PDF) [file pone.0287233.s003.pdf]

**S2 Table** Characteristics of the 360 fever in neutropenia (FN) episodes studied

| Patient characteristics | n (%) |
|---|---|
| Gender | |
| - Female | 173 (48%) |
| - Male | 187 (52%) |
| **Malignancy related characteristics** | |
| Type of malignancy | |
| - Acute lymphoblastic leukemia | 177 (49%) |
| - Acute myeloid leukemia | 16 (4%) |
| - Hodgkin lymphoma | 8 (2%) |
| - Non-Hodgkin lymphoma | 42 (12%) |
| - Central nervous system tumor | 32 (9%) |
| - Other solid tumor | 85 (24%) |
| Relapsed malignancy | 21 (6%) |
| Bone marrow involvement | 24 (7%) |
| **Therapy related characteristics** | |
| Chemotherapy intensity | |
| - 1 | 27 (8%) |
| - 2 | 276 (77%) |
| - 3 | 39 (11%) |
| - 4 | 18 (5%) |
| Central venous access device | 358 (99%) |
| **Clinical characteristics at presentation** | |
| - Temperature at presentation >= 39°C | 172 (48%) |
| - Severely reduced general condition | 52 (14%) |
| **Hematological characteristics at presentation** | |
| - Hemoglobin < 90g/l (n=358) | 214 (60%) |
| - Leucocyte count < 0.3 G/l | 124 (34%) |
| - Absolute neutrophil count < 0.1 G/l (n=252) | 138 (55%) |
| - Absolute monocyte count < 0.1 G/l (n=242) | 166 (69%) |
| - Thrombocyte count < 50 G/l (n=258) | 190 (53%) |
| **Outcomes** | |
| - Bacteremia | 56 (16%) |
| - Serious medical complication | 30 (8%) |
| - Safety relevant event | 72 (20%) |
| - Severe sepsis | 22 (6%) |
| - Intensive care unit admission | 16 (4%) |
| - Death | 0 (0%) |

NOTE: if not otherwise indicated characteristics were known in all 360 episodes.
