# Supplementary material for: Outcome prediction in pediatric fever in neutropenia: Development of clinical decision rules and external validation of published rules based on data from the prospective multicenter SPOG 2015 FN definition study
Source: PLoS One. 2023 Aug 2;18(8):e0287233. doi: 10.1371/journal.pone.0287233 (PMC10395874; doi:10.1371/journal.pone.0287233)
Supplement: S3 Table — *result of two level mixed regression due to model failure of the three-level mixed model. Abbreviations: CI, confidence interval; OR, Odds ratio; FN, fever in neutropenia; FN-BACT, fever in neutropenia with bacteremia. (PDF) [file pone.0287233.s004.pdf]

S3 Table Association of characteristics with bacteremia, serious medical complications and safety relevant events, univariable analysis

Outcomes	Bacteremia (n=56)			Serious medical complication (n=30)			Safety relevant event (n=72)		
	Univariable mixed logistic regression			Univariable mixed logistic regression			Univariable mixed logistic regression		
Characteristics	N	OR (95% CI)	p-value	N	OR (95% CI)	p-value	N	OR (95% CI)	p-value
Patient related characteristics									
Sex									
Male	37	Reference	-	15	Reference	-	44	Reference	-
Female	19	0.49 (0.25-0.96)	0.038	15	1.01 (0.41-2.48)	0.98	28	0.6 (0.31-1.17)	0.133
Age group at screening									
1 to 4 years	25	Reference*	-	10	Reference	-	31	Reference	-
5 to 8 years	10	0.48 (0.21-1.07)*	0.072	9	1.04 (0.35-3.14)	0.942	16	0.59 (0.26-1.32)	0.199
9 to 12 years	7	0.91 (0.35-2.38)*	0.845	5	1.66 (0.42-6.48)	0.468	7	0.73 (0.25-2.11)	0.558
≥13 years	14	1.33 (0.6-2.92)*	0.481	6	1.33 (0.33-3.92)	0.844	18	1.42 (0.61-3.33)	0.418
Malignancy related characteristics									
Type of malignancy				Model failure					
Acute lymphoblastic leukemia	32	Reference	-	20	-	-	43	Reference*	-
Acute myeloid leukemia	10	7.55 (2.56-22.29)	<0.001	3	-	-	10	5.45 (1.45-20.44)*	0.012
Hodgkin lymphoma	1	0.65 (0.08-5.45)	0.689	0	-	-	1	0.43 (0.05-4.07)*	0.461
Non-Hodgkin lymphoma	6	0.79 (0.29-1.94)	0.560	3	-	-	7	0.67 (0.25-1.82)*	0.435
Central nervous system tumor	1	0.15 (0.02-1.11)	0.063	2	-	-	3	0.29 (0.08-1.1)*	0.068
Other solid tumor	6	0.34 (0.14-0.86)	0.022	2	-	-	8	0.33 (0.13-0.8)*	0.015
Relapsed malignancy	4	1.58 (0.43-5.82)	0.496	1	0.48 (0.05-4.99)	0.541	5	1.52 (0.42-5.47)	0.519
Therapy related characteristics									
Chemotherapy intensity (expected duration of neutropenia)									
1 (no neutropenia expected)	4	1.2 (0.38-3.83)	0.753	3	1.34 (0.3-5.9)*	0.701	5	0.96 ()0.3-3.05*	0.951
2 (≤10 days expected)	35	Reference	-	21	Reference*	-	49	Reference*	-
3 (>10 days expected)	15	4.52 (1.99-10.27)	<0.001	5	1.96 (0.53-7.23)*	0.311	15	3.47 (1.35-8.94)*	0.01
4 (myeloablative therapy)	2	0.71 (0.15-3.31)	0.659	1	0.66 (0.07-5.98)*	0.710	3	0.65 (0.16-2.69)*	0.550
Central venous access device	56	Model failure	-	30	Model failure	-	72	Model failure	-

Bone marrow involvement	10	5.97 (2.01-17.76)	0.001	1	0.45 (0.05-3.76)*	0.461	10	4.2 (1.44-12.23)	0.009
Time since diagnosis > 1 month	35	0.43 (0.21-0.88)	0.021	25	1.81 (0.64-5.15)	0.263	50	0.65 (0.33-1.27)	0.206
Prior episode of FN	30	0.91 (0.49-1.67)	0.759	18	1.18 (0.52-2.64)*	0.693	41	1.09 (0.62-1.04)	0.76
Prior episode of FN-BACT	10	0.3 (0.05-1.78)	0.186	9	2.52 (1.09-5.82)	0.031	16	0.91 (0.29-2.86)	0.873
Clinical characteristics at presentation									
Autumn-winter season	29	1.02 (0.55-1.88)	0.947	15	0.93 (0.41-2.12)	0.869	38	1.09 (0.61-1.93)	0.774
Presentation out of office time	25	0.64 (0.34-1.21)	0.17	14	0.74 (0.33-1.68)	0.475	34	0.72 (0.4-1.3)	0.279
Temperature ≥ 39°C	22	0.7 (0.37-1.3)	0.255	10	0.49 (0.21-1.16)*	0.105	27	0.62 (0.34-1.11)	0.108
Severely reduced general condition	20	4.78 (2.38-9.58)	<0.001	14	6.99 (2.95-16.55)	<0.001	26	5.81 (2.83-11.95)	<0.001
Systemic inflamamatory response syndrome	47	1.51 (0.65-3.53)	0.341	26	2.03 (0.62-6.64)	0.241	62	1.79 (0.78-4.09)	0.169
Hematological characteristics									
Hemoglobin < 90g/l	38	1.72 (0.9-3.27)*	0.098	14 (n=29)	0.59 (0.26-1.37)	0.222	44 (n=71)	1.33 (0.76-2.34)*	0.314
Leucocyte count <0.3G/l	33	3.47 (1.83-6.58)	<0.001	15	2.03 (0.96-4.3)*	0.065	39	3.25 (1.69-6.24)	<0.001
Absolute neutrophil count <0.1G/l	13 (n=24)	1.32 (0.57-3.1)	0.519	5 (n=14)	0.43 (0.08-2.29)	0.323	16 (n=32)	0.93 (0.23-3.75)	0.922
Absolute monocyte count <0.1G/l	20 (n=24)	3.19 (1.05-9.7)	0.041	11 (n=13)	4.55 (0.35-59.88)	0.249	25 (n=31)	2.45 (0.78-7.64)	0.124
Platelet count <50G/l	40	2.49 (1.52-4.08)	<0.001	23	3.21 (1.29-8.01)	0.012	52	2.8 (1.52-5.18)	0.001

*result of two level mixed regression due to model failure of the three level mixed model.

Abbreviations: CI, confidence interval; OR, Odds ratio; FN, fever in neutropenia; FN-BACT, fever in neutropenia with bacteremia
