# Supplementary material for: Outcome prediction in pediatric fever in neutropenia: Development of clinical decision rules and external validation of published rules based on data from the prospective multicenter SPOG 2015 FN definition study
Source: PLoS One. 2023 Aug 2;18(8):e0287233. doi: 10.1371/journal.pone.0287233 (PMC10395874; doi:10.1371/journal.pone.0287233)
Supplement: S4 Table — Abbreviations: CDR, clinical decision rule; CI, confidence interval; DD, derivation dataset; ICU, intensive care unit; LR, low risk; NPV, negative predictive value; PPV, positive predictive value; TN, true negative; TP, true positive; VD, validation dataset; rVD, restricted validation dataset. Bold = reproducibility criteria fulfilled for sensitivity / specificity. (PDF) [file pone.0287233.s005.pdf]

S4 Table Results of internal crossvalidation of the new CDRs and external validation of published CDRs

									Low vs intermediate/high risk						Complete rule			
									Sensitivity		Specificity							
Rule	*Outcome*	*Episodes*	*Outcome, n (%)*	*LR, n (%)*	*True pos*	*True neg*	*False pos*	*False neg*	*% (95% CI)*	*Diff from Derivation, % (P value)*	*% (95% CI)*	*Diff from Derivation, % (P value)*	*PPV, % (95% CI)*	*NPV, % (95% CI)*	*Median AUC ROC (95% CI)*	*Median Spec at Sens 95%*	*Median Spec at Sens 90%*	*Median Spec at Sens 80%*
New CDR 2021	bacteremia	360	56 (15.6)	99 (27.5)	54	97	207	2	96.4 (86.6-99.4)		31.9 (26.8-37.5)		20.7 (16-26.2)	98 (92.2-99.6)	0.813	0.350	0.447	0.642
New CDR 2021 crossvalidated internally			56 (15.6)	99 (27.5)	51	94	210	5	91.1 (85.7-92.9)	- 5.3 (0.244)	30.9 (28.9-33.2)	- 1 (0.750)	19.5 (18.8-20)	95 (92.4-96)				
New CDR 2021	serious medical complications	360	30 (8.3)	151 (41.9)	28	149	181	2	93.3 (76.5-98.8)		45.2 (39.7-50.7)		13.4 (9.2-18.9)	98.7 (94.8-99.8)	0.690	0.118	0.236	0.458
New CDR 2021 crossvalidated internally			30 (8.3)	149 (41.4)	28	147	181	2	93.3 (93.3-93.3)	0 (1)	44.8 (44.8-44.8)	- 0.4 (0.942)	13.4 (13.4-13.4)	98.7 (98.7-98.7)				
New CDR 2021	safety relevant event	360	72 (20)	98 (27.2)	67	93	195	5	93.1 (83.7-97.4)		32.3 (27-38.1)		25.6 (20.5-31.4)	94.9 (87.9-98.1)	0.799	0.286	0.393	0.616
New CDR 2021 crossvalidated internally			72 (20)	88 (24.4)	66	82	206		91.7 (87.5-93.1)	- 1.4 (0.839)	28.5 (25.7-30.9)	- 3.8 (0.174)	24 (23.2-24.7)	92.8 (89.7-93.9)				
DD-Rackoff 1996	bacteremia	115	24 (20.9)	19 (16.5)	24	19	72	0	100 (86-100)		20.9 (13.8-30.3)		25 (17-35.1)	100 (79.1-100)	NA			
VD-Rackoff		242	36 (14.9)	76 (31.4)	32	72	134	4	88.9 (73-96.4)	- 11.1 (<0.001)	35 (28.5-41.9)	+ 14.1 (<0.001)	19.3 (13.7-26.3)	94.7 (86.4-98.3)	0.558	0.160	0.310	0.396
rVD-Rackoff		166	20 (12)	57 (34.3)	18	55	91	2	90 (66.9-98.2)	- 10 (<0.001)	37.7 (29.9-46.1)	+ 16.8 (<0.001)	16.5 (10.3-25.1)	96.5 (86.8-99.4)	0.581	0.195	0.367	0.424
DD-Klaassen 2000	significant bacterial infection	227	43 (18.9)	83 (36.6)	36	76	108	7	83.7 (70-91.9)		41.5 (34.3-48.7)		25 (18.3-33)	91.6 (82.9-96.3)	NA			
VD-Klaassen		242	45 (18.6)	76 (31.4)	37	68	129	8	**82.2 (67.4-91.5)**	**- 1.5 (0.947)**	**34.5 (28-41.7)**	**- 7 (0.055)**	22.3 (16.4-29.5)	89.5 (79.8-95)	0.570	0.097	0.194	0.359
rVD-Klaassen		182	29 (15.9)	65 (35.7)	24	60	93	5	**82.8 (64.4-93)**	**- 0.9 (1)**	**39.2 (24.1-47.5)**	**- 2.3 (0.623)**	20.5 (13.8-29.2)	92.3 (82.2-97.1)	0.605	0.118	0.235	0.411
DD-Baorto 2001	bacteremia	1171	189 (16.1)	164 (14)	179	154	828	19	94.7 (90.5-97.1)		15.7 (13.5-18.1)		17.8 (15.5-20.3)	93.9 (88.8-96.9)	NA			
VD-Baorto		269	44 (16.4)	56 (20.8)	41	53	172	3	**93.2 (80.3-98.2)**	**- 1.5 (0.91)**	23.6 (18.3-29.8)	+ 7.9 (0.002)	19.2 (14.3-25.3)	94.6 (84.2-98.6)	0.584	0.179	0.262	0.344
rVD-Baorto		220	33 (15)	46 (20.9)	31	44	143	2	**93.9 (79.4-98.6)**	**- 0.8 (1)**	23.5 (17.8-30.4)	+ 7.8 (0.004)	17.8 (12.6-24.5)	95.7 (84-99.2)	0.587	0.199	0.2767	0.349

									Sensitivity		Specificity							
Rule	*Outcome*	*Episodes*	*Outcome, n (%)*	*LR, n (%)*	*True pos*	*True neg*	*False pos*	*False neg*	*% (95% CI)*	*Diff from Derivation, % (P value)*	*% (95% CI)*	*Diff from Derivation, % (P value)*	*PPV, % (95% CI)*	*NPV, % (95% CI)*	*Median AUC ROC (95% CI)*	*Median Spec at Sens 95%*	*Median Spec at Sens 90%*	*Median Spec at Sens 80%*
DD-Madsen 2002	bacteremia	157	12 (7.6)	81 (51.6)	11	80	65	1	91.7 (64.6-98.5)		55.2 (47-63.1)		14.5 (7.8-24.8)	98.8 (92.4-99.9)	NA			
VD-Madsen		227	29 (12.8)	145 (63.9)	15	131	67	14	51.7 (32.9-70.1)	- 40 (<0.001)	66.2 (59.1-72.6)	+ 11 (0.002)	18.3 (10.9-28.7)	90.3 (84-94.4)	0.598	0.069	0.137	0.274
rVD-Madsen		161	17 (10.6)	106 (65.8)	10	99	45	7	58.8 (33.5-80.6)	(<0.001)	68.8 (60.4-76.1)	(0.001)	18.2 (9.5-31.4)	93.4 (86.4-97.1)	0.641	0.083	0.165	0.331
DD-Rondinelli 2006	severe infection complications	283	93 (32.9)	NA	NA	NA	NA	NA	NA		NA		NA	NA	NA			
VD-Rondinelli		358	69 (19.3)	26 (7.3)	65	22	267	4	94.2 (85.1-98.1)		7.6 (4.9-11.5)		19.6 (15.5-24.3)	84.6 (64.3-95)	0.543	0.068	0.126	0.239
rVD-Rondinelli		115	17 (14.8)	4 (3.5)	17	4	94	0	100 (77.1-100)		4.1 (1.3-10.7)		15.3 (9.4-23.7)	100 (39.6-100)	0.503	0.110	0.175	0.301
DD-Ammann 2010	adverse events	423	122 (28.2)	165 (39)	112	155	146	10	91.8 (85.6-95.5)		51.5 (45.9-57.1)		43.3 (37.3-49.7)	93.9 (88.8-96.9)	NA			
VD-Ammann		357	130 (36.4)	167 (46.8)	77	114	113	53	59.2 (50.3-67.7)	- 32.6 (<0.001)	**50.2 (43.5-56.9)**	**- 1.3 (0.749)**	40.5 (33.5-47.9)	68.3 (60.6-75.1)	0.563	0.068	0.174	0.248
rVD-Ammann		304	106 (34.9)	142 (46.7)	67	103	95	39	63.2 (53.2-72.2)	-28.5 (<0.001)	**52 (44.8-59.1)**	**+ 0.5 (0.940)**	41.4 (33.8-49.4)	72.5 (64.3-79.5)	0.613	0.099	0.213	0.315
DD-Hakim 2010	invasive bacterial infection or culture negative sepsis	323	47 (14.6)	223 (69)	35	211	65	12	74.5 (60.5-84.8)		76.4 (71-81.1)		35 (25.9-45.3)	94.6 (90.6-97.1)	0.776 (0.7-0.85)			
VD-Hakim		252	45 (17.9)	188 (74.6)	21	164	43	24	46.7 (31.9-62)	- 27.8 (<0.001)	**79.2 (73-84.4)**	**+ 2.8 (0.381)**	32.8 (21.9-45.8)	87.2 (81.4-91.5)	0.631	0.122	0.182	0.387
rVD-Hakim		81	11 (13.6)	63 (77.8)	5	57	13	6	45.5 (18.1-75.4)	- 29 (0.062)	**81.4 (70-89.4)**	**+ 5 (0.395)**	27.8 (10.7-53.6)	90.5 (79.8-96.1)	0.672	0.134	0.320	0.409
DD-Suttitossatam 2020	severe adverse outcome	95	11 (11.5)	NA	7	NA	NA	4	63.6 (31.6-87.6)		NA							
VD-Suttitossatam		360	28 (7.8)	260 (72.2)	10	242	90	18	35.7 (19.3-55.9)	- 27.9 (0.004)	72.9 (67.7-77.5)		10 (5.2-18)	93.1 (89.1-95.7)	0.543	0.056	0.113	0.226
rVD-Suttitossatam		355	25 (7)	256 (72.1)	9	240	90	16	36 (18.7-57.4)	- 27.6 (0.008)	72.2 (67.5-77.4)		9.1 (4.5-17)	93.8 (89.9-96.3)	0.544	0.057	0.113	0.226
DD-Haeusler 2020	bacteremia	858	111 (12.9)	84 (9.8)	108	81	666	3	97.3 (91.7-99.3)		10.8 (8.6-13.3)		14 (11.6-16.6)	96.4 (89.2-99.1)				
VD-Haeusler		358	56 (15.6)	20 (5.6)	54	18	284	2	**96.4 (86.6-99.4)**	**- 0.9 (1)**	6 (3.7-9.4)	- 4.8 (0.009)	16 (12.3-20.4)	90 (66.9-98.2)	0.659	0.087	0.170	0.337
rVD-Haeusler		353	52 (14.7)	20 (5.6)	50	18	283	2	**96.2 (85.7-99.3)**	**- 1.1 (0.935)**	6 (3.7-9.5)	- 4.8 (0.009)	15 (11.4-19.4)	90 (66.9-98.2)	0.665	0.088	0.182	0.370

DD-Haeusler 2020	likely bacterial infection	858	198 (23.1)	84 (9.8)	189	75	585	9	95.5 (91.3-97.8)		11.4 (9.1-14.1)		24.4 (21.5-27.6)	89.3 (80.2-94.7)	0.64 (0.60-0.68)			
VD-Haeusler		358	110 (30.7)	20 (5.6)	106	16	232	4	**96.4 (90.4-98.8)**	**+ 0.9 (0.836)**	6.5 (3.9-10.5)	- 4.9 (0.019)	31.4 (26.5-36.6)	80 (55.7-93.4)	0.592	0.085	0.150	0.281
rVD-Haeusler		353	105 (29.7)	20 (5.7)	101	16	232	4	**96.2 (90-98.8)**	**+ 0.7 (0.916)**	6.5 (3.9-10.5)	- 4.9 (0.019)	30.3 (25.5-35.6)	80 (55.7-93.4)	0.596	0.083	0.152	0.290
DD-Haeusler 2020	ICU admission	858	24 (2.8)	84 (9.8)	24	84	750	0	100 (82.8-100)		10.1 (8.2-12.4)		3.1 (2-4.6)	100 (94.6-100)				
VD-Haeusler		358	16 (4.5)	20 (5.6)	15	19	323	1	93.6 (67.7-99.7)	- 6.4 (<0.001)	5.6 (3.5-8.7)	- 4.5 (0.007)	4.4 (2.6-7.4)	95 (73.1-99.7)	0.567	0.088	0.180	0.372
rVD-Haeusler		353	13 (3.7)	20 (5.7)	12	19	321	1	92.3 (62.1-99.6)	- 7.7 (<0.001)	5.6 (3.5-8.7)	- 4.5 (0.008)	3.6 (2-6.4)	95 (73.1-99.7)	0.596	0.038	0.131	0.341

Abbreviations: CDR, clinical decision rule; CI, confidence interval; DD, derivation dataset; ICU, intensive care unit; LR, low risk; NPV, negative predictive value; PPV, positive predictive value; TN, true negative; TP, true positive; VD, validation dataset; rVD, restricted validation dataset.

Bold = reproducibility criteria fulfilled for sensitivity / specificity
